# An Important Clue in the Sonographic Diagnosis of Internal Carotid Artery Agenesis: Ipsilateral Common Carotid Artery Hypoplasia

**DOI:** 10.1155/2014/516456

**Published:** 2014-07-02

**Authors:** Omer Kaya, Cengiz Yilmaz, Bozkurt Gulek, Gokhan Soker, Gokalp Cikman, Ibrahim Inan, Selahaddin Demirduzen

**Affiliations:** ^1^Department of Radiology, Adana Numune Teaching and Research Hospital, 01240 Adana, Turkey; ^2^Department of Radiology, Namik Kemal University, 59100 Tekirdag, Turkey; ^3^Department of Radiology, School of Medicine, Cukurova University, 01330 Adana, Turkey; ^4^Department of Radiology, Medeniyet University, Goztepe Teaching and Research Hospital, 34730 Istanbul, Turkey; ^5^Department of Ophthalmology, Adana Numune Teaching and Research Hospital, 01240 Adana, Turkey

## Abstract

A 42-year-old female patient, who had been diagnosed with an occlusion of her left internal carotid artery (ICA) following Doppler ultrasonographic (US) and digitally-subtracted angiographic (DSA) examinations performed in an outer healthcare center in order to eliminate the underlying cause of her complaint of amorosis fugax, later applied to our hospital with the same complaint. At Doppler US performed in our hospital's radiology department, her right common carotid artery (CCA) was normal, but her left CCA was hypoplastic. The right internal artery (ICA) was validated as normal. At the left side, however, the ICA was apparent only as a stump and it did not demonstrate a continuity. The diagnosis of ICA agenesis was confirmed by the utilization of Doppler US, CT, and DSA imaging, and it was concluded also that ipsilateral CCA hypoplasia could be evaluated as an important clue to the diagnosis of ICA agenesis.

## 1. Case Report

**Figure 1:**
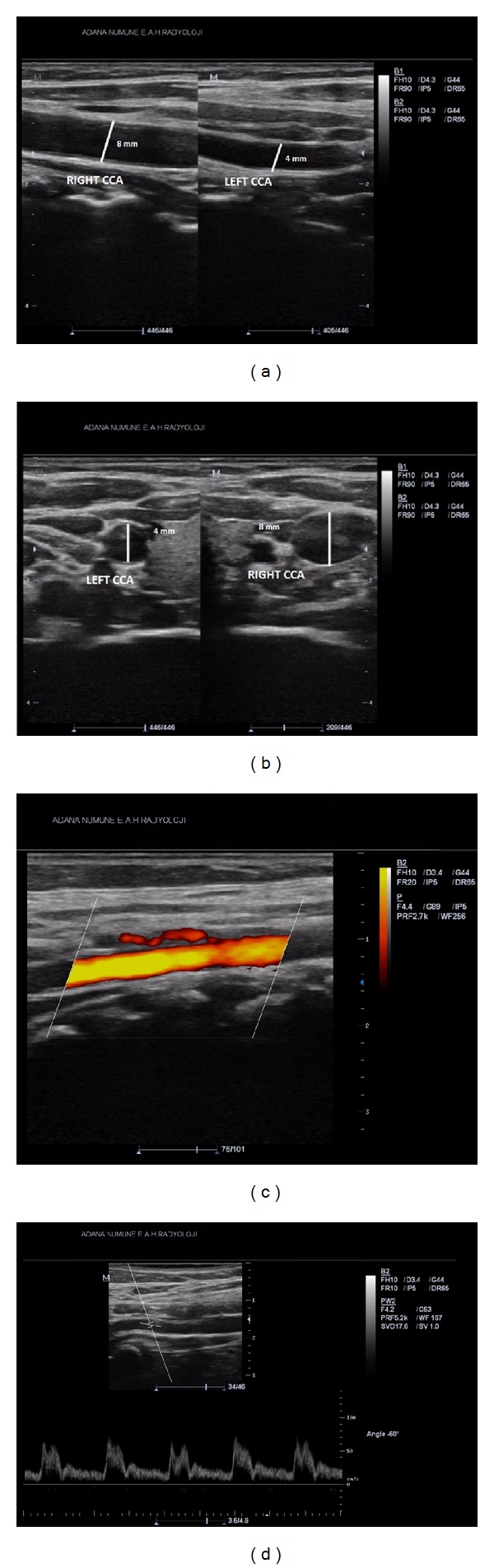
(a) Doppler US images demonstrate the diameters of the right and left CCA, which are 8 mm and 4 mm, in respect. (b) These axial Doppler US images reveal the diameters of the right and left ICA. (c) The Doppler view of the left ECA and its branching. (d) The spectral drawing demonstrating that the vessel mentioned in Figure 1(c) is indeed the ECA.

**Figure 2:**
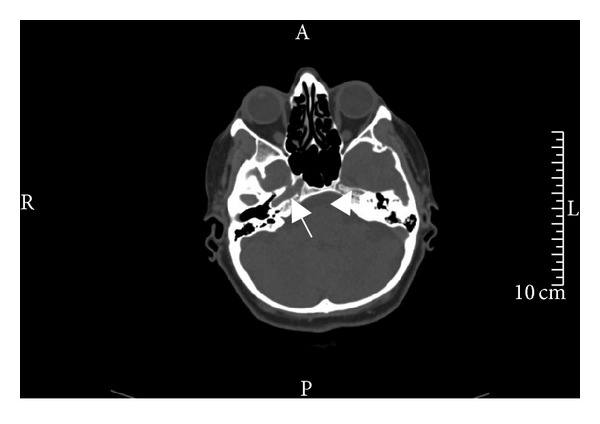
This axial CT image of the cranial base shows that while the carotid canal in the petrosal bone is clearly visible on the right side (arrow), there is no visibility of a left carotid canal (arrow head).

**Figure 3:**
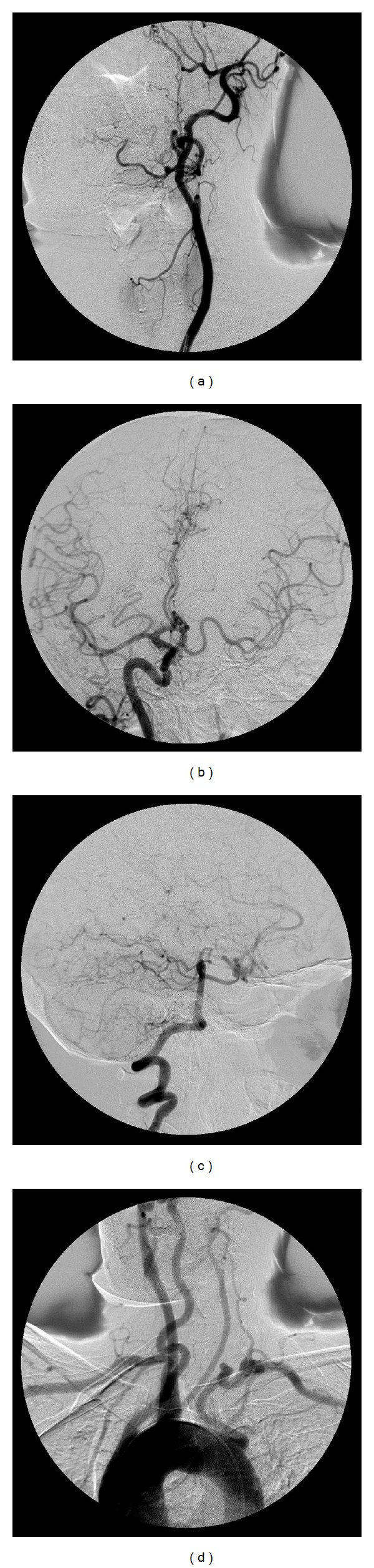
(a) After contrast injection to the left common carotid artery, the presumed (but absent) left ICA tract does not reveal any filling, while the left ECA and its branches are clearly visible. (b) At this Towne projection image, the filling of the left anterior cerebral and medial arteries via the patent anterior communicating artery is seen. (c) At this right lateral projection, vascular fillings of the left hemicranium with contrast via the posterior communicating artery following the injection of the vertebral artery are seen. (d) At arcus aortography, the right vertebral artery is seen as the first branch stemming from the aortic arch. The left common carotid artery caliber is substantially decreased. The left vertebral and subclavian arteries look tortuous.

A 42-year-old woman applied to the ophthalmology department with a complaint and history of a half-hour-long visual loss of her left eye one day ago. At clinical examination, both of her eyes were found to have full vision, and also microscopic fundus examinations revealed no pathological conditions. The patient gave a history of a prior application to an outer medical center with the same complaint, and there she had been diagnosed with a left ICA occlusion, following Doppler US and angiographic examinations. The patient applied to our department with the demand of an extracranial carotid Doppler US examination. At Doppler US, the right CCA diameter was found to be 8 mm, whereas the left one came out to be 4 mm (Figures 1(a) and 1(b)). The right CCA bifurcation and the ICA and ECA were found to be normal. At the left side, spectral data for the ECA (Figure 1(d)) and the particular branching which demonstrates that the vessel is ECA indeed were present, whereas there was no Doppler signal and gray-scale findings for the ICA (Figure 1(c)). Because the left CCA was hypoplastic and there were no findings of atherosclerotic plaques in other arterial segments; it was thought that the left ICA might not be suffering an occlusion at all, but instead it might well be agenetic. Based on the presumption of an ICA agenesis, a computed tomographic (CT) examination of the skull base was performed. CT images revealed a normally appearing right carotid canal but no clear image of the left one (Figure 2). Then, the previous angiographic images of the patient were reevaluated, and it was then recognized that the left ECA and its branches were well visible, while the left ICA and its branches did not show any filling with contrast (Figure 3(a)). It was also noted that the filling of the left anterior and medial cerebral arteries was sustained by the patent anterior communicating artery following the injection of contrast into the right carotid artery (Figure 3(b)) and also via the posterior communicating artery following the injection of the vertebrobasilary system (Figure 3(c)). In addition to the ICA agenesis, another surprising finding was that the right vertebral artery was not stemming from the right subclavian artery but instead was originating from the aortic arch, as its first branch (Figure 3(d)). It was finally understood that the patient did not have an occlusion of the left ICA, but instead her left ICA was agenetic. She also had an additional aortic arch anomaly. At the end, it was concluded that an ipsilateral CCA hypoplasia might be taken as a valuable clue in the differential diagnosis of ICA agenesis and occlusion.

## 2. Discussion

Agenesis of the internal carotid artery system is a rare entity [1], and its incidence is lower than 0.01% [2]. It is thought that a unilateral ICA agenesis is due to an intrauterine mechanical or hemodynamic stress, in a way similar to the unilateral rotation and wrapping of the embryo by an amniotic band at the 4th–8th gestational weeks [2]. The cause of the bilateral ICA agenesis, on the other hand, is yet to be discovered [3].

Sonographic findings suggestive of ICA agenesis include nonvisualization of the the carotid bifurcation, ipsilateral hypoplasia of the CCA, and a normal ipsilateral CCA Doppler waveform [1, 4]. At DSA, nonvisualization of the ICA shortly after its origin and an enlarged ECA may be seen [5]. The most common type of collateral flow is through the circle of Willis. However, to a rare extent, collateral flow is provided via persistent embryonic vessels or from transcranial collaterals originating from the external carotid artery (ECA) system [2].

An ICA agenesis may be misdiagnosed as an occlusion as was in our case [4]. In the setting of a differential diagnosis in ICA occlusion, the imaging of the skull base and the investigation of the presence of the carotid canal are mandatory [2]. This is because the development of the ICA takes place only secondarily to the development of the carotid canal [6], and this points to the fact that, in the absence of a carotid canal, the ICA has not developed [7]. On the other hand, as was in our case, an ipsilateral CCA hypoplasia too is an important finding in the diagnosis of an ICA agenesis. In our case, the right CCA diameter was 8 mm, while the left one was found to be only 4 mm, thus pointing to its hypoplastic situation (Figures 1(a) and 1(b)).

The differentiation of ICA agenesis from occlusion is rather important because of the increase in the risk of the presence of an aneurysm in association with the agenesis, in comparison to the normal population [8]. The incidence of an intracranial aneurysm in the normal population is 2–4%, while it is reported to be as high as 24–67% in the presence of agenesis or aplasia [7, 8]. Usually, aneurysms of the Willis circle accompany ICA agenesis and intracranial aneurysm leading to intracranial bleeds have been detected in 25% of symptomatic ICA agenesis cases [7, 9–11]. Arachnoid cysts accompanying ICA agenesis may be present in some cases [12].

ICA agenesis may show an association with other different vascular anomalies of the aortic arch [13, 14]. This was the situation in our case, who had a right vertebral artery coming out from the aortic arch as its first branch, rather than the right subclavian artery (Figure 3(d)).

In case of an ICA agenesis, neurologic deficits are usually absent or minimized, due to the collateral circulation which saves the situation [1]. Collaterals are formed by the ICA and the vertebrobasilary system via the circle of Willis, together with the anastomoses which stem from the ipsilateral ECA [9, 15]. Still, in some cases, an association with a transient ischemic attack (TIA) has been demonstrated [16]. Other symptoms include headache, loss of hearing, visual loss, and situations of hemiparesis which are or are not accompanied by cranial nerve palsies [8, 17].

## 3. Results

It is essential to visualize the carotid canal in order to make a proper decision in the differential diagnosis of ICA agenesis and occlusion. Unilateral CCA hypoplasia is an important clue in the diagnostic process.

ICA agenesis is usually asymptomatic due to the presence of collaterals. But because the incidence of intracranial aneurysms is in an increase, the risk of intracranial bleeding is in an increase as well, and this is why the differentiation between an occlusion and agenesis of the ICA has become of utmost importance. Besides, association with certain symptoms such as headache, hearing and visual loss, and cranial nerve palsies may also be present.

Another important fact is that ICA agenesis may be together with other vascular anomalies of the aortic arch. Due to this reason, other vascular structures at the aortic arch and in the carotid system must also be demonstrated.
